# Hospital and Institutional News

**Published:** 1919-01-04

**Authors:** 


### HOSPITAL AND INSTITUTIONAL NEWS.

### THE LATE SIR ERNEST TRITTON.

The Metropolitan Hospital Sunday Fund has experienced a sad loss through the death of Sir Ernest Tritton, Vice-Chairman of the Fund since 1914, member of the Finance Committee from its foundation, and Chairman of it since October 1911. Sir Ernest was one of the four trustees of the Herring Estate, and his services in connection with its taking over proved invaluable. The work involved was heavy, and its transaction required financial knowledge of a high order. The securities handed over had to be realised, which, thanks to Sir Ernest and the Finance Committee, was accomplished very much to the advantage of the Fund. In 1914, the year when Sir Ernest became Vice-Chairman of the Fund, the total receipts were £64,000. In 1918 they had increased to £92,000, a record sum directly attributable to the interest Sir Ernest Tritton took in the Fund and his continuous work for it. In 1914 the Sunday Fund exhibited a tendency to decline, a fact which would have prevented most men from accepting the Vice-Chairmanship. Not so Sir Ernest Tritton, who welcomed it as an opportunity to render strenuous personal service through the Fund to the hospital, and the figures just quoted show the magnificent results which have accrued. Sir Ernest Tritton had a personal charm, and his continuous and untiring labours in the interest of the Hospital Sunday Fund, and in other matters to which he lent a hand, have won for him a host of friends who will never fail to remember him and his services with deep gratitude and thankfulness. The officers of the Fund testify that they could never go to him at any time, however busy he was, or whatever the time of year, that they did not find him perfectly prepared, with the best of grace, to put aside the whole of his own business affairs and immediately absorb himself in the work of the Hospital Sunday Fund. A regular attendant at the meetings, the members of its committees will miss the presence of Sir Ernest Tritton so long as they themselves are associated with the work in which the late Vice-Chairman took so profound an interest. It will be difficult to find a successor to fill the vacant offices, but we have confidence that the example and work of the late Sir Ernest Tritton will stir up some of his late colleagues to fill the vacancy and continue the good work with undiminished energy and increasing success. Here, indeed, is an opportunity for a good man and zealous worker.

### AN INSPIRING EXAMPLE TO FOLLOW.

It is a fact, remarkable as it is welcome, that in the first year of Sir Ernest Tritton's assumption of office the working expenses of Hospital Sunday in London were reduced by nearly £500 as compared with the previous year. In other words, the percentage of working expenses to the gross receipts fell from 4.745 per cent. in 1913 to 3.952 per cent. in 1914, the average percentage since the establishment of the Fund in 1873 being then 3.945. Thanks largely to the late Sir Ernest Tritton, the Metropolitan Hospital Sunday Fund has to-day attained an efficiency of administration and a greatly larger income, despite the fact that the increased yield has been brought about in the four years of the great war. During those four years the assiduous devotion and constant work of our late friend and colleague, Sir Ernest Tritton, steadily reduced the working expenses of the Fund to 3.503 of the gross receipts, as against an average percentage since the establishment of the Fund in 1873 of 3.896. Further, the new system so heartily adopted by the clergymen and ministers who have put their heart into the work, and directed many collections by bands of workers from people who are not in the habit of attending places of worship, as well as from the members of their congregation, has increased the yield from London collections on Hospital Sunday in 1918 by 50 per cent. compared with the receipts in the year when the late lamented Sir Ernest Tritton assumed the office of vice-chairman. Who will take his place and go and do likewise?

### NEW YEAR HONOURS.

Among the names appearing in the first instalment of the New Year Honours published on January 1 are the following: Dr. William Leslie Mackenzie, M.D., Medical Member of the Local Government Board for Scotland, and Mr. George Danvers Thane, LL.D., F.R.C.S., Principal Inspector under Cruelty to Animals Act, Home Office, on whom the honour of Knighthood has been conferred. Dr. R. Bruce Low, M.D., Assistant Medical Officer Local Government Board (retired), has been appointed a Companion of the Order of the Bath, in recognition of services during the war, and the honour of Knight Bachelor has been conferred on Lieut.-Col. John Hewat, M.B., Assistant Director of Medical Services of the Union of South Africa. Amongst the Naval Honours the names of the following medical men appear: *C.B.* (*Civil Division*), Surgeons Captains Walter Godfrey Axford, R.N., and Arthur Stanley Fanee, R.N.; *K.C.M.G.*, Surgeon Rear-Admiral George Welch, C.B.; *C.M.G.*, Surgeons Commanders Robert Dundonald Jameson, R.N., and Hugh Somerville Burniston, M.B., R.N.; *O.B.E.* (*Military Division*), Surgeon-Lieutenant A. E. Iles, M.B., F.R.C.S., R.N., Lieutenant-Commander H. Isherwood, R.N.V.R., Surgeon-Commander (Acting Surgeon Captain) M. L. Penny, R.N., Surgeon-Lieutenant Commander H. D. Drennan, M.B., B.A., R.N., *K.C.V.O.*, Sir George Anderson Critchett, C.V.O.

### THE RED CROSS DIARIST.

It is to be hoped that as full a record as possible will be made of the voluntary work done by members of the British Red Cross during the war. The ordinary official survey, as was proved even in Mr. Laurence Binyon's heroic attempt to give form and proportion to the doings of the British Red Cross in France, must be too sketchy or too statistical. The field to be surveyed is too large for one writer. On the other hand, many, very many, people must have carried their diaries into hospital and camp, canteen, and ambulance. Cannot these be recovered? The quality of the work so far written has been poor in the extreme. Cannot something be done to collect and edit the diaries? This is the idea of Colonel Williams Endicott, of the American Red Cross in Great Britain, who has sent the following request to every member of the American Red Cross in his "command": "You may have kept a diary; if so, will you not read it over and copy out all the portions (amplifying them where necessary)" which bear on the individual experience of the writer. Will these diaries ever see the light?

### PHOTOGRAPHS OF ARCHANGEL.

A word of notice is due to the first official photographs of the American Red Cross expedition at Archangel, which appeared in the *Bulletin* of December 18. It looks a dreary place enough, and the photograph of the street, so deep in mud that a raised gangway has to be built down the middle, is a picture of northern desolation indeed. From this it is a relief to turn to the news in the same paper which states that American hospitals in this country are emptying so fast that Salisbury Court, Southampton, and the two military hospitals at Liverpool will be the only American hospitals in Great Britain to remain open during the winter.

### A WELL-MERITED INCREASE OF SALARY.

Lambeth Borough Council have decided to increase the salary of Dr. Joseph Priestley, Medical Officer of Health, to £1,000 per annum, subject to the approval of the Local Government Board. Dr. Priestley has held the appointment twenty-three years, his initial salary under the old Lambeth Vestry being £700 a year, but on the constitution of the borough it was increased to £800, whilst in 1909 he was granted a further increase of £100 so long as he should carry out certain bacteriological examinations for the council. Dr. Priestley based his application for an increase of salary on the grounds of the increased cost of living and heavy Income Tax, his length of service with the council, the nature of his duties and the fact that he was the only chief officer or employee who had not received an increase to their salary during these hard times. The council did not haggle about granting this additional £100 to Dr. Priestley, whose length of service fully justified the increase.

### A CHILD'S FEAR OF THE DARK.

Time was when the correspondence columns of our contemporary *The Spectator* were often concerned with instances of animal intelligence, a fact which sometimes provoked the mirth of the irreverent. Lately, however, an excellent topic has been started, namely the phenomenon that the young child of the present day is by no means so much frightened of the dark as were his parents. One reason advanced in explanation of this is that the hymns and moral instruction generally for children were formerly of a rather terroristic nature, whereas nowadays we are a good deal less superstitious. On reflection, there would seem to be much truth in this contention. "If I die before I wake" and "In the long night watches, angels round my head" are—well, not to give offence, let us say, rather too pan-animistic for the lively imagination of children at a tender and helpless age. But whatever the explanation, this writer can confirm from personal observation the assertion of *The Spectator's* lady correspondent that modern children are no more afraid of the dark regions upstairs than modern adults are; while the fact that the children he has in mind have not been taught as were those of the last generation goes to confirm also her theory—which indeed had already suggested itself to him before reading her interesting communication.

### PUBLIC DENTAL CLINICS.

The president of the Central Counties branch of the British Dental Association, in his address at the recent annual meeting of that body, declared that the Ministry of Health would certainly take steps to give efficient dental treatment to the masses. The chief obstacle was the paucity of dentists: even if all the unregistered practitioners were included there would still be a great shortage for the undertaking of a complete service. The reason of this shortage was the unsatisfactory legal position at present of the dental profession, whereby a qualified man, who had to spend much time and money on his examinations, and pass strict examinations, had to meet the competition of the unregistered. Until this disability was removed, qualified dentists would remain scanty. As to public dental clinics, he advised their being established in all thickly populated centres under the Public Health Department, but worked by a Dental Committee, and financed under the National Insurance Scheme. There should be a permanent staff of registered dentists, nurses, and mechanics, and a visiting one of anæsthetists and local registered dentists, all paid. To increase the numbers of qualified dentists it was urged that the curriculum should be shortened and made less costly.

### TUBERCULOSIS HOSPITAL AND THE LAW.

The law's delays are so great, especially just now, that probably our readers will have forgotten all about the case of Frost *v.* National Memorial Association for the Prevention, Treatment and Abolition of Tuberculosis. Briefly a hospital for surgical tuberculosis was "summoned" by its neighbours for being a nuisance. The upshot was that it cleared its character, but that its assailants established that restrictive covenants did exist affecting the property (which had been given to the hospital) and that thereby the use of the house for the above purpose was not strictly allowable. Delay of judgment for the "duration" and for six months afterwards, was however granted. The defendants appealed, but lately an arrangement has been come to, and Cardigan House will be turned into a tuberculosis dispensary with quarters for the tuberculosis officer when the six months are up. Unfortunately the defendants have to pay costs, such costs to be taxed by the District Registrar at Newport. We wish more power to the District Registrar's elbow.

### PROVISION FOR TUBERCULOUS CHILDREN.

On December 10 last the London County Council unanimously adopted an outline scheme whereby provision was planned for 3,600 places in open-air schools; the capital outlay being estimated at £30 per place, and the annual cost of maintenance at a trifle over £20, of which latter figure half would be recoverable by way of Government grant. The Education Committee contemplated schools of about 100 children each, but the Finance Committee thought larger ones could be run on more economical lines. Excepting that open-air education favours the survival of some constitution-less children, whose decease might be a favourable event eugenically speaking, it is hard to see how money could be spent better in an anti-tuberculosis campaign than by beginning with the rising generation and implanting in them a sense of proper domestic hygiene and ventilation.

### THE DISMISSAL OF A CHAPLAIN.

The Portsmouth Board of Guardians, after having failed to persuade the chaplain, the Rev. W. H. Skryne, to resign, has decided by a narrow majority to give him one month's notice from January 1. the reason given in the resolution is that he has "failed satisfactorily to perform his duties." Mr. Skryne refused to resign on the ground that he is a permanent official who could only be asked to resign with the consent of the Local Government Board. He added that if he was considered after a thorough investigation, not to have carried out the rules, he would then resign. He reminded the Guardians that he has not had the use of a chapel for religious services and that this had made his work difficult. Since the majority in favour of his dismissal was only that of eight votes to six, it is still not too late, we hope, to hold the investigation which Mr. Skryne has invited and to avoid friction or lengthy appeals to Whitehall.

### A FACTORY SURGEON ON THE HOUSING QUESTION.

On Tuesday last Dr. R. C. Brown, of Preston, resigned his appointment as certifying surgeon under the Factory Act after holding it for 55 years. In 1863 the western district of Preston was assigned to him, and ten years later he was transferred to the eastern district. The Factory Act of 1863 applied chiefly to the cotton industry, but when it became the "Factory and Workshop Act" its application was general. Moreover, the changes which Dr. Brown has helped to enforce include the raising of the age at which children were allowed to begin work in the factories from eight to nine years in 1872, to ten in 1874, and to twelve in 1878. At thirteen still the whole day may be spent in the factory provided that a certain number of school attendances each year for five years have taken place. The home visiting undertaken by Dr. Brown in his capacity of medical officer convinced him that though the girls in poor families do much of the housework and some of the cooking, when they married, which was often early, they seemed to have no systematic knowledge of laundry, cooking, or household management. After a report on the subject, Dr. Brown's views were generally shared, and now in Preston every girl of eleven attending an elementary school is required to receive domestic instruction at one of the three centres in the town. Dr. Brown believes that the housing problem demands such instruction if the new houses are to be made into decent homes.

### THE SUPPLY OF CREAM AFTER JANUARY 6.

To relieve the present shortage of milk, the Food Controller has issued a new Order, the Cream Order, 1918, which places further restrictions on the supply of cream. The Order will come into force on January 6 next, and will further restrict the sale of cream. Cream for children under five will be limited to a maximum of half a pint per week. To obtain cream for such children applications should be made to the local Food Committee for a permit which should be lodged with the retailer. In the case of invalids, permits for cream may be granted as an alternative to butter and margarine. These will be granted by the Food Committee on a medical certificate in the case of invalids suffering from certain specified diseases. Applications in respect of invalids suffering from other diseases must be submitted by the Food Committee for special authorisation from the Medical Section of the Ministry of Food. Such applications must be accompanied by a medical certificate giving specific reasons why cream is recommended. Directions as to cases in which applications for cream may be entertained by Food Committees are being furnished to medical practitioners for their guidance. Applications in respect of inmates of institutions must be made by the Head of the institution on their behalf.

### HOSPITALS' SUCCESSFUL LEGAL ACTION.

Some curious and unusual wording in the will of the late Miss Elizabeth Daubeny led to an action in the Chancery Division which was recently decided by Mr. Justice Lawrence. Miss Daubeny, wishing to benefit the Bristol Royal Infirmary and the Cheltenham General Hospital, left them, to quote the words of the will, "the residue of my property (to the amount of £200)." The residue now amounted to about £2,000, and the question that the Court had to decide was whether the institutions should share the whole of that amount or were limited to £200 between them, or whether they should receive £200 each. The judge held that the words in parentheses "(to the amount of £200)" were merely an estimate of the extent of the residue. He accordingly made a declaration to that effect, and ordered that the whole of the residue be divided in equal shares between the two hospitals, the costs to come out of the residue.

### THE PRICE OF EGGS.

The Council of the National Utility Poultry Society views with great concern the "severe injury" done to the poultry-keeper by reason of the existing orders made by the Ministry of Food. The maximum retail price of the fresh home-produced egg is now fixed at 5½d., which is the same price chargeable for the imported (so-called) "fresh" egg. The Board of Agriculture and Fisheries was advised by its own Poultry Advisory Committee, after "thoroughly going into the cost of production," that the current price should not be less than 6d., it being shown that even at that price the egg could not be sold to yield any profit to a large number of producers. In fixing the price of the egg, it is urged that inadequate steps were taken to fix the prices of the foodstuffs necessary for its production, with a result that the British poultry-keeper, who has to buy all his food, is in a very critical position. In one instance it is said that the poultry-keeper is forced to pay to-day £62 for a ton of damaged rice as against £25 for rice fit for human consumption. Had the prices of poultry foodstuffs been adequately controlled by the Ministry of Food, the Council declares that there would not have been such a shortage of eggs, and the present necessity for importing the millions of doubtful Egyptian eggs would not have arisen. As the value of the British poultry industry has been officially estimated at over £30,000,000 per annum, harsh treatment of an important branch of "home" food production must ultimately diminish the egg supply of this country.

### A GRIEVANCE REMEDIED.

From time to time the Lambeth Board of Guardians have had to complain that expectant mothers, not residents in Lambeth, who had made arrangements in advance to be admitted to the General Lying-in Hospital, York Road, were, on their arrival, informed that they could not be admitted as there were no vacant beds, and the patients were referred to the Lambeth Infirmary without, however, ascertaining if they could be received. The Guardians have received a letter from Miss R. E. Whyte, secretary of the hospital, stating that the committee regretted the difficulties that had arisen, but promised to take preventive measures in the future. Each intending patient is to be recommended to telephone to the hospital before leaving home for admission to ascertain whether a bed is available at the time.

### THE EMPTY STOCKING.

Just as an enterprising railway company adorns its stations with two maps in which the shape of Cornwall and Italy are compared, to the advantage of the "Cornish Riviera," so Sergeant Noel Irving has found a capital subject for a cartoon for the Christmas number of the *Gazette* of the 3rd London General Hospital, in the fact that the territory over which the Allies advanced before the armistice, from Nieuport to near the Scheldt, from Lille to Tournai, from La Fère to Sedan, and from St. Mihiel to Rethel, resembles a leg. But he calls it "the empty stocking," beside which little boy Prussia and his little sister Austria stand in tears. This is the best invention of the Christmas number. Before the armistice the writers of the *Gazette* used to wonder when its last issue would occur. Now the editor still invites subscriptions for six months: the humorist! The hospital's work is not yet done. And those in the army of occupation hoping for their discharge have to remember that the General Staff still has its anxieties, because the knock-out blow smashes the fabric of government as well as that of the army, and until the stability of the former is guaranteed we have to police those whom we have conquered.

### THE ORIGIN OF CHRISTMAS CUSTOMS.

In so far as we use holly, mistletoe, and the Dragon bowl we are all Druids at Christmas if those who study the origin of these customs are to be believed. Yule himself was a god of the Saxons where sacred fire was kept burning by the priests from year to year when they kindled his log from which other household fires were lighted. We summarise these statements from the *Gazette* of the 4th Southern General Hospital, Plymouth; but the truth seems to be that annual festivals are older than the oldest recorded religion, and that when one superseded another it took over the old festivals and placed its own interpretation upon them. The fact that the same customs and the same myths are found in different parts of the world seems to show that the human race has certain elemental aspirations, and the similarity of the different rituals argues the depth in which these are rooted in mankind. The gods change their names, but they, like the saturnalia and the seasons, return and are welcomed; for if the god Yule had never been worshipped a great fire in midwinter would be worshipped still.

### THE GERMAN RAILWAY SYSTEM.

The latest triumph of the Army has been to send trains into the centre of Prussia to bring back prisoners and wounded. Cologne, the railway centre on which the enemy's main armies chiefly relied, has at last, therefore, been put to a useful and proper purpose, and Red Cross trains are the first sent by the Allies. The suffering of the prisoners thus rescued seem in no way to have been diminished by the armistice, and deaths are reported to have been high in several of the camps visited.

### THIS WEEK'S DRUG MARKET.

The market has not yet been fully re-opened and the stock-taking process is still going on. It is not anticipated that business will be active for several weeks. In the meantime prices in many instances are merely nominal, and until the market is in full swing again it will be difficult to form a definite idea as to the probable trend of prices. Generally speaking, however, it would seem fairly safe to assume that whatever else happens, values will not tend upwards, although, of course, there may be exceptions. Since last report the prices of several articles have been re-adjusted on a lower level, and it is not improbable that before the New Year is far advanced the values of some of these will be still lower. Uncontrolled potassium bromide is distinctly cheaper; this is not altogether surprising. Antifebrin is lower. Paraldehyde has had a further fall in price and the tendency in the price of phenacetin is downwards. Imported saccharin is offered at lower rates.

